# Novel Vertical 3D Structure of TaO_x_-based RRAM with Self-localized Switching Region by Sidewall Electrode Oxidation

**DOI:** 10.1038/srep21020

**Published:** 2016-02-17

**Authors:** Muxi Yu, Yimao Cai, Zongwei Wang, Yichen Fang, Yefan Liu, Zhizhen Yu, Yue Pan, Zhenxing Zhang, Jing Tan, Xue Yang, Ming Li, Ru Huang

**Affiliations:** 1 Institute of Microelectronics, Peking University, Beijing 100871, China

## Abstract

A novel vertical 3D RRAM structure with greatly improved reliability behavior is proposed and experimentally demonstrated through basically compatible process featuring self-localized switching region by sidewall electrode oxidation. Compared with the conventional structure, due to the effective confinement of the switching region, the newly-proposed structure shows about two orders higher endurance (>10^8^ without verification operation) and better retention (>180h@150 °C), as well as high uniformity. Corresponding model is put forward, on the base of which thorough theoretical analysis and calculations are conducted as well, demonstrating that, resulting from the physically-isolated switching from neighboring cells, the proposed structure exhibits dramatically improved reliability due to effective suppression of thermal effects and oxygen vacancies diffusion interference, indicating that this novel structure is very promising for future high density 3D RRAM application.

Flash is the mainstream of non-volatile memory (NVM), which is widely used in the field of embedded system and applied electronics. However, as the integration density continues rising, flash is reaching the physical limitation due to reliability issues as well as process cost. Thus various emerging memories with new storage mechanisms were put forward as candidates for flash replacement, such as ferroelectric random access memory (FeRAM), magnetic random access memory (MRAM), phase change random access memory (PRAM), resistive random access memory(RRAM) and so on^1^,^2^,^3^,^4^,^5^,^6^. Among them, RRAM attracts arising attentions due to its advantages of simple structure, excellent performance and outstanding scaling capability^6^,^7^,^8^. Recently, RRAM has been officially listed in International Technology Roadmap for Semiconductors (ITRS) as one of the most promising emerging memories in future^9^.

Resistive switching occurs in a wide range of materials among which the binary transition metal oxides (TMO) facilitate the simple fabrication process and stable structure, thus gained extensive attention. A large variety of candidate metal-oxide materials have been demonstrated for RRAM technology, such as TaO_x_^10^,^11^,^12^, HfO_x_^13^,^14^, TiO_x_^15^, AlO_x_^16^, CuO_x_^17^, and so on. Among them, TaO_x_ -based RRAM has shown superior merits such as low operation voltage^18^, fast switching speed^19^, high thermal stability^20^, distinctive retention capability^21^,^22^, excellent uniformity^23^,^24^, as well as the process compatibility^25^, especially, the most prominent feature of TaO_x_ -based RRAM is the extremely high endurance due to the simple thermodynamic equilibrium phases of Ta-O systems^26^,^27^,^28^,^29^, which paves the way for reliable storage application.

As mentioned above, in order to satisfy the exponentially grown needs for big-data storage, the integration density of storage system is continuously enlarging while the size of storage cell is continuously scaling. Although a high-density 16 Gb RRAM with 27 nm Technology has been recently reported^30^, the further shrinking of 2D RRAM size, especially in sub-10 nm era, strongly relies on complex processes and advanced lithography technology, facing the cost and fabrication challenges^31^, which hinders RRAM as replacement of NAND flash memory for low-cost mass storage. Therefore, several 3D RRAM structures have been presented aiming to competing with 3D NAND technology in the future^32^,^33^,^34^. Wherein compared with horizontal 3D structure that simply stacking 2D RRAM cells layer by layer which inevitably increasing cost of patterning^35^, vertical 3D RRAM, especially vertical 3D TMO-based RRAM (TMO VRRAM) has been considered as a more promising candidate for future ultra-high density non-volatile memory applications in terms of the bit-cost scalability, high switching speed and low power consumption^36^,^37^.

Nowadays the researches on 3D RRAM mainly focus on the scalability of both vertical and horizontal dimensions to increase the storage capability, and sub-nm sidewall electrode are demonstrated in different researches^38^,^39^. However, the accompanying reliability problems demand much more consideration, especially for the additional dimension in 3D RRAM compared with 2D structure. To be specific, the reported TMO VRRAMs based on transition metal oxides so far, usually have a whole continuous metal oxide layer formed along the sidewall of the hole etched through multiple stacks^36^,^37^,^38^,^39^, which may cause reliability degradation originated from oxygen vacancies diffusion along this continuous oxide layer, particularly when stacks are scaled to extremely small dimensions. One typical example is that disturbance may occur to the vertical adjacent unselected cell when operating the selected cell, which may even cause function disability^40^.

It is widely accepted that the resistive switching of RRAM is strongly correlated to the defects modification in switching oxide; specifically, the movement of oxygen ions plays a decisive role in characteristics of TMO-based RRAM devices^1^,^2^,^3^. Therefore, unwanted diffusions of oxygen ions in switching layer will have serious impacts on the reliability and performance, or even functions of RRAM^41^,^42^. In this regard, effective isolation of vertical adjacent cells is critical to cut off unwanted diffusion paths of oxygen ions, and then restrain the performance degradation. In 2D RRAM, several solutions are put forward to reduce the unwanted oxygen vacancies lateral diffusion, including cutting off the continuous metal oxide layer by etching and introducing encapsulated cell structure by patterning^20^,^43^,^44^,^45^. However, these approaches are not practical for 3D VRRAMs for the reason that the continuous metal oxide layer along the sidewall cannot be easily patterned and selectively etched.

In this work, a novel 3D vertical RRAM structure was proposed and successfully demonstrated by two-layer stacked 3D TaO_x_ RRAM. The unique difference is that in the proposed structure, thermal oxidation is adopted to form the self-localized switching cells (TaO_x_) which is only located on the separated sidewall electrode (Ta) layer, naturally isolated by the undisturbed isolation layer. While in conventional structure, atomic layer deposition (ALD) or physical vapor deposition (PVD) may unavoidably form the whole continuous resistive switching film, which also exists besides the isolation layer. Therefore, the simply change of the switching layer formation process by sidewall electrode oxidation (SEO) effectively cuts off the vertical adjacent resistive switching layers without increasing process complexity or consuming additional area, demonstrating dramatic reliability improvements and scaling capability of the proposed structure.

## Results and Discussion

### A. Sidewall electrode oxidation optimization

According to the previous reports^46^,^47^,^48^, the properties of tantalum oxide formed by Ta oxidation mainly depend on oxidation process conditions, especially on oxidation temperature and time: below 400 °C, most of the oxygen is dissolved in the tantalum lattice (which is called the solution of interstitial oxygen)^46^, while above 400 °C, the oxidation rate becomes faster as the annealing temperature rises^47^; additionally, after the complete oxidation, the thickness of the oxidized part would double^48^.

**Figure 1:**
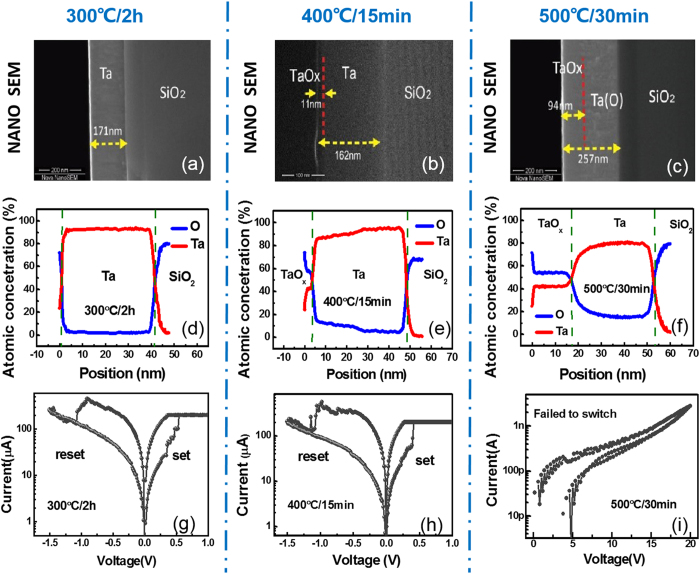
SEM cross-section images (Top-(**a–c**)); AES profile (Middle-(**d–f**)); and corresponding device switching characteristics (Bottom-(**g–i**)) of sidewall electrode oxidation experiments under different annealing conditions: annealing 2 h/300 °C (Left-(**a,d,g**)); annealing 15 min/400 °C (Middle-(**b,e,h**)); annealing 30 min/500 °C (Right-(**c,f,i**)). The thickness of oxidized TaO_x_ layers increase with elevated temperature.

As in our device, since the switching material is formed through oxidation of Ta sidewall electrode, Ta oxidation condition and optimization are critical to the proposed structure. Therefore, three annealing conditions (300 °C/2 h, 400 °C/15 min, 500 °C/30 min) were investigated with comprehensive consideration of temperature and time to fabricate 2D RRAM devices with simply metal-insulator-metal (MIM) structure (Ta/TaO_x_/Pt) (See Oxidation Optimization in Methods section), allowing for both the component analysis as well as corresponding electrical measurements (as shown in Fig. 1).

The combination of cross-sectional profiles provided by scanning electron microscope (SEM) images (Fig. 1(a–c)) and oxygen distributions along depth direction provided by auger electron spectroscopy (AES) (Fig. 1(d–f)) clearly show the different composition distributions in three annealing conditions. Among which, 400 °C/15 min annealing is enough to form 11 nm TaO_x_ film with proper oxygen concentration gradient, demonstrating that the novel critical process of this novel structure meets the temperature requirement of CMOS back-end process. In addition, according to the DC measurement displayed in Fig. 1 (g–i), device annealed under 400 °C/15 min condition shows best switching characteristics. In comparison, 500 °C/30 min annealing devices cannot be switched due to the 94 nm thick oxide layer, and in case of 300 °C/2 h annealing samples, although there are some lucky devices can also be switched (on/off ratio smaller than 400 °C/15 min ones), their oxide thickness can hardly be detected (as depicted in Fig. 1(a,d)), indicating the risk of reproducibility and yield.

### B. Novel 3D vertical RRAM fabrication

**Figure 2:**
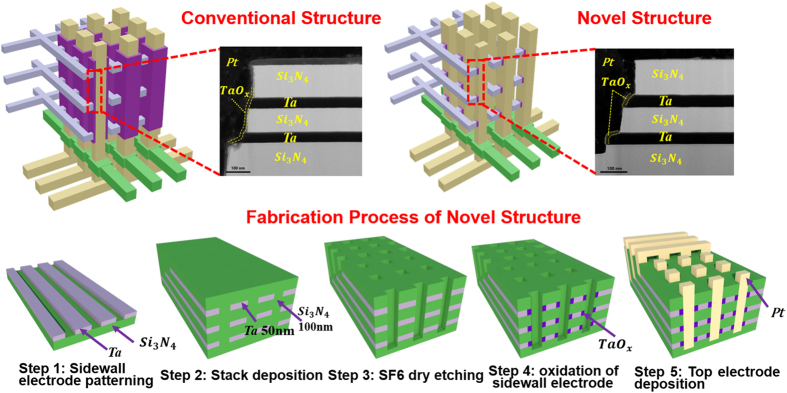
(Top) 3D vertical RRAM array architecture of conventional structure with continuous oxide resistive switching layer, and novel structure with self-localized resistive switching region formed around Ta sidewall electrode. TEM images of enlarged view of RRAM cells in both structures demonstrate the differences. (Bottom) Fabrication process of the proposed novel 3D vertical RRAM structure.

**Figure 3:**
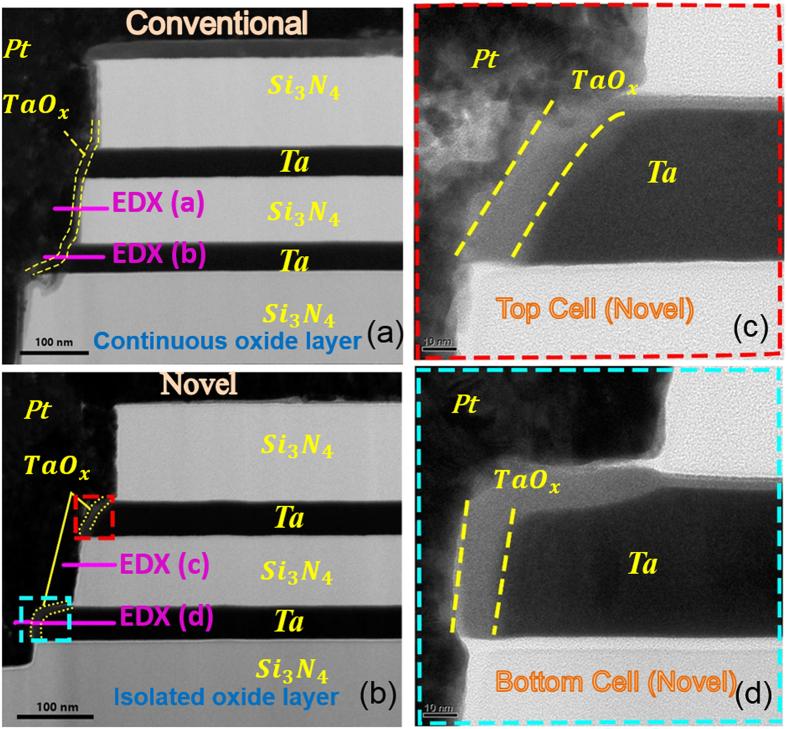
TEM images of (**a**) conventional structure; (**b**) proposed novel structure; (**c**) top cell in the proposed novel structure; (**d**) bottom cell in the proposed novel structure. TEM images clearly show that the switching layers of top cell and bottom cell are a consecutive TaO_x_ film in conventional structure while the switching regions are isolated by Si_3_N_4_ layer and confined to the Ta sidewall electrodes. The proposed SEO structure is well demonstrated here.

**Figure 4:**
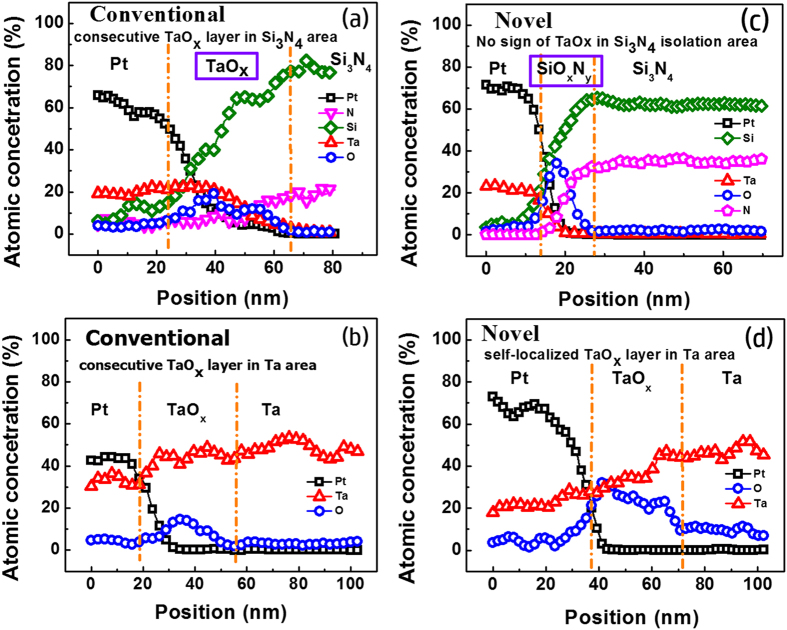
(Left) Horizontal EDX line scans with corresponding elements profiles labeled as (**a,b**) in Fig. 3(a); (Right) Horizontal EDX line scans with corresponding elements profiles labeled as (**c,d**) in Fig. 3(b). EDX analysis of elements profile further confirms the switching region in the proposed SEO structure is totally isolated by Si_3_N_4_ layer.

By utilizing the above optimized condition, the novel 3D VRRAM with cells in two vertical stacked layers is experimentally demonstrated based on Ta sidewall electrode oxidation, adopting 400 °C/15 min oxidation condition. Meanwhile, devices with conventional 3D structure are also fabricated as a comparison (See Device Fabrication in Methods section). The schematic view of both structures and the detailed fabrication processes of the proposed novel 3D RRAM cell are illustrated in Fig. 2. The core distinction of the fabrication processes of both structures lies in that, instead of PVD or ALD to form a continuous switching layer in conventional structure, partially oxidation of the isolated sidewall electrodes can naturally form the self-confined switching areas on the edge of sidewall electrodes in the novel 3D structure. The transmission electron microscope (TEM) and energy dispersive x-ray spectroscopy (EDX) characterizations confirm the differences as shown in Figs 3 and 4.

TEM images display that the spontaneously confined TaO_x_ switching regions only exist around the sidewall electrodes after annealing in the proposed novel structure (Fig. 3(a)), while a consecutive TaO_x_ switching layer deposited by sputtering covers on the whole sidewall of the hole-region in conventional structure (Fig. 3(b)). To further carefully compare the elements profile differences between both structures, EDX component analysis are conducted on the isolation layer and sidewall electrode region near sidewall of the hole in both structures. Shown by the Fig. 4(a,b), TaO_x_ can be seen only in the resistive cell region in the novel structure, and no TaO_x_ in the counterpart of the isolation layer, while TaO_x_ exists in both cell and isolation layers in conventional structure (Fig. 4(c,d)). Additionally, in conventional structure cell region shown by Fig. 4(a), it is observed that element N is detected in the TaO_x_ region besides the isolation Si_3_N_4_ layers, indicating that a conducting TaN phase may be formed by reaction between Ta_2_O_5_ and Si_3_N_4_, raising the risk of short-circuiting the vertical adjacent cells. By contrast, the cells in novel structure are cut off completely without the similar concerns, further demonstrating the improvements.

It is worth noting that the concentration gradient in novel 3D structure is similar to that of the optimization experiment in Fig. 1, which validates that our self-confined 3D structure will not affect the oxidation process to form TaO_x_ with uniform quality which can ensure the resistive switching properties. In addition, the enlarged TEM images (Fig. 3c,d) indicate that switching layer thicknesses of top and bottom cells are also similar to the 2D cell with the same optimized annealing condition, which further indicates the stability and uniformity of the critical oxidation process. This feature is essential for RRAMs to maintain highly uniform performance in 3D era with scaled dimensions. For instance, as the increase of stacking layers and the decrease of hole dimensions, the conformality of TMO switching layer deposition (PVD or ALD) process would inevitably get deteriorated due to the limitation of conventional etching and deposition processes, leading to the severe variations of deposited switching layer thickness and consequently resulting in electrical performance dispersions in conventional 3D VRRAM devices. However, in case of the proposed structure, the thickness of switching TMO layer is mainly determined by partially oxidation of sidewall electrodes, which is not sensitive to the scaled dimension of 3D structure, facilitating the uniform formation of switching cells and accordingly the good device-to-device uniformity control.

### C. Switching characteristics

Electrical characterizations were performed on Agilent B1500A semiconductor characterization system. During the measurements, the voltage is applied on Ta sidewall electrode, while keeping Pt top electrode grounded all the time.

**Figure 5:**
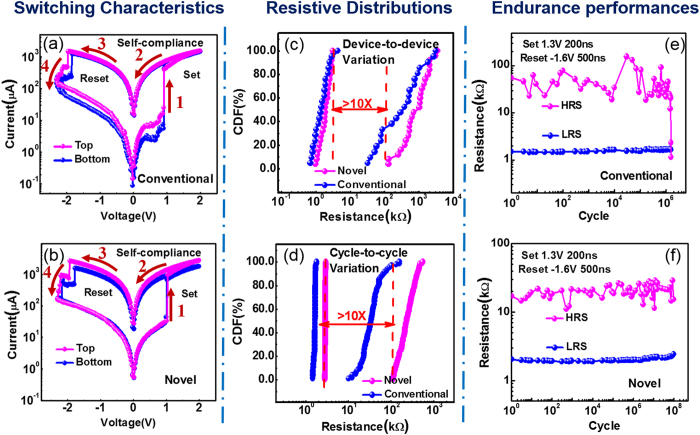
(Switching Characteristics) Typical I-V curves from randomly chosen adjacent cells in both conventional structure (**a**) and proposed SEO structure (**b**). The devices show not only excellent bipolar switching characteristics but also nearly identical initial switching curves. (Resistive Distributions) Resistive distributions in conventional structure and proposed SEO structure. (**c**) device-to-device variability obtained from 30 randomly selected devices; (**d**) cycle-to-cycle variability obtained from 10^7^ consecutive pulse operation cycles. HRS/LRS ratio >10X is obtained in proposed SEO structure. (Endurance performances) Endurance characteristics of conventional structure and proposed SEO structure. Pulse operation configuration: set 1.3 V/200 ns; reset −1.6 V/500 ns. ~10^8^ cycles was obtained without verification and any noticeable degradation in proposed SEO structure. Conventional structure degrades after ~10^6^ cycles.

The typical switching characteristics of novel structure and conventional structure are displayed in Fig. 5(a,b), respectively. The resistance switching occurs along direction of the arrows shown in the figures, with set voltage about 1V and reset voltage about 2V in both structures, which meets the requirement of integration. Meanwhile, the very similar performances demonstrate that the switching layer formed by optimized oxidation process in novel structure exhibits the comparable switching characteristics with the sputtering switching layer in conventional structure, which further confirms the TEM and EDX results in Figs 3 and 4. In addition, the identical characteristics of the up- and bottom- cell in the proposed structure complementally verifies the process uniformity of sidewall electrode oxidation (SEO), corresponding to the same thicknesses in both layer shown in Figs 3 and 4. Furthermore, switching window >10X was obtained without requiring current compliance in reset operation, which is beneficial to simplify the peripheral circuit design. The excellent self-compliance and controllable reset process are due to the gradient profile of oxygen concentration in TaO_x_ corresponding to the SEO process.

Since that the distributions of resistance are critical parameters for memory array performance and also key issues for 3D RRAM array integration, 30 randomly selected devices were measured to obtain the cumulative probability of both structures, as well, cycle-to-cycle variability was obtained from 10^7^ consecutive pulse operation cycles in one randomly selected device of both structures, respectively. Compared with conventional structure, remarkable improvements of both device-to-device (Fig. 5(c)) and cycle-to-cycle (Fig. 5(d)) uniformity of HRS are achieved in the novel structure. As mentioned above, the SEO process helps to form uniform oxide films in different layers, thus improving the device-to-device uniformity. At the same time, the enhanced cycle-to-cycle uniformity is mainly benefited from the confined switching region which can effectively suppress the diffusion of oxygen vacancies (V_O_s) and consequently improving the stability of the conductive filaments (CFs) morphology.

### D. Reliability behaviors

The comparison of reliability performances (endurance and retention) was also experimentally performed in detail. During pulse measurements, the proper pulse programming conditions are optimized as: 1.3 V/200 ns for set operation and −1.6 V/500 ns for reset operation without any verification operation. Endurance as high as ~10^8^ cycles was obtained without any noticeable degradation in the proposed cell structure (Fig. 5(e)). In contrast, (Fig. 5(f)) shows that the HRS of the conventional cell degraded at only ~10^6^ cycles under the same test configuration. The marvelous improvement of endurance capability of the novel 3D structure is attributed to the more confined oxygen distribution of physically isolated resistive cells due to SEO process. To be specific, in the novel structure, benefited from the good isolation, oxygen vacancies (V_O_s) involved in the formation or rupture of CFs in the cell region can be well maintained as the cycling times increasing, thus significantly mitigating the endurance degradation.

**Figure 6:**
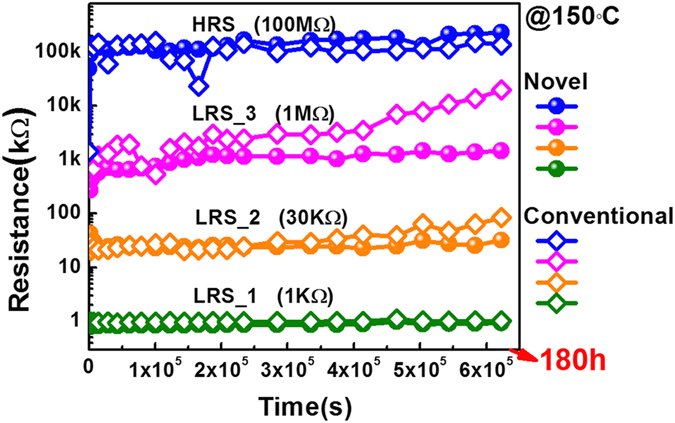
Retention behavior of four different resistive states (LRS_1 (10^3 ^ohm), LRS_2 (3*10^4 ^ohm), LRS_3 (10^6 ^ohm), HRS (10^8 ^ohm)) at 150 °C for conventional structure and proposed SEO structure. For conventional structure, resistance state shows distinct drift to higher resistance state. For proposed SEO structure, each resistance state can maintain almost unchanged >180 h@150 °C.

As for the non-volatile memory (NVM), retention property determines the time limit for data storage, which is the critical reliability parameter. In the case of RRAM, the degradation of low resistive state (LRS) is more serious, since that the CFs evolution in LRS is directly related to the V_O_s diffusions under the action of both concentration gradient and thermal effect. Especially in small dimensions, the trade-off of retention and low operation current gets more obvious^41^, for the reason that the CFs are more vulnerable in the low current region and small sizes. This degradation will be more serious in 3D structures as the heat accumulation is more obvious due to the heat dissipation problems of the complex multi-layer structures. In this paper, in order to carefully investigate the influence of 3D structures on the retention performances as well as the V_O_s diffusion behavior, devices of four different resistance states (LRS-1, LRS-2, LRS-3 & HRS) of both structures were baked at 150 ^o^C. As shown in Fig. 6, both low resistive state (LRS) and high resistive state (HRS) of conventional devices exhibit distinct drift to higher resistance states. And notably, the aggravation of retention degradation with increased LRS in conventional devices well demonstrates the trade-off between retention capability and low current operation in conventional 3D structure^41^. However, only slight changes in all the four resistive states were observed in the novel structure devices (Fig. 6) even after the 180 h baking, indicating the superior retention feature of the proposed structure. Furthermore, the proposed structure, with good stability of relative high resistance in LRS, also demonstrates great potential for low power application.

**Figure 7:**
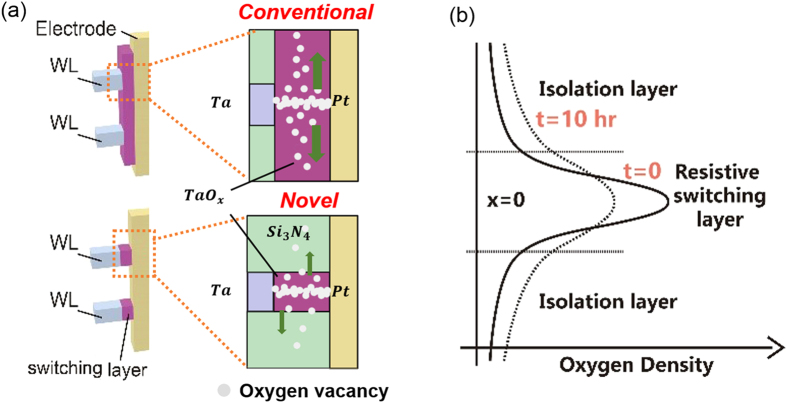
Schematic diagram of (**a**) Oxygen vacancies diffusion inside the conventional structure and the proposed SEO structure; (**b**) thermal diffusion model of the time dependent oxygen vacancies density distribution.

We believe that the significant reliability improvements of the proposed sidewall electrode oxidation (SEO) cell are due to the automatic formation of self-confined switching area which can significantly inhibit the degradation caused by the combination of oxygen vacancies diffusion and thermal effect during switching process. Additionally, adoption of Si_3_N_4_ dielectric layers, which acts as powerful oxygen-blocking layers and heat sinks^43^,^44^,^45^, further prevents the unwanted diffusions of oxygen vacancies. It is widely accepted that CF consisting of V_O_s in TMO RRAM device has higher V_O_ concentration in the center of CF and the redistribution of V_O_s driving by concentration gradient and thermal effect will give critical impacts on resistance drift of LRS and consequently retention characteristics of RRAM devices^21^,^22^. Figure 7(a) illustrates the schematic diagram of oxygen vacancies diffusions inside the proposed SEO cell and the conventional cell. A thermal diffusion model was proposed to gain insightful understanding of the reliability issues (Fig. 7(b)), based on which distribution of V_O_s density in the resistive cell region versus time can be calculated.

Corresponding to Fig. 7(b), the vertical direction in 3D structure is set as X axis to calculate the diffusion of V_O_s between resistive cell and the adjacent isolation layer or resistive film in both structures. The processes of V_O_s concentration varying with time in the resistive cell regions can be described by the following equations^21^,^22^:

### V_O_s concentration


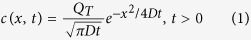


In which, *c*(*x*, *t*) is the time-varying V_O_s concentration along the cell region, *Q*_*T*_ is the total quantity of oxygen vacancies per unit area, *D* is the diffusion coefficient with the expressions as follows:

### Diffusion coefficient


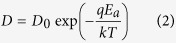


### Intrinsic diffusion coefficient


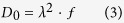


where *E*_*a*_ is the activation energy (1.16 ev for TaO_x_^21^ and >4.7 eV^49^ for Si_3_N_4_), *k* is the Boltzmann constant (1.38 × 10^−23^ J/K), *q* is the electron charge (1.6 × 10^−19^C), λ is the lattice constant in isolation layer (0.76 for TaO_x_ and 0.79 for Si_3_N_4_), *f* is the oscillation frequency of phonon (~10^13^).

**Figure 8:**
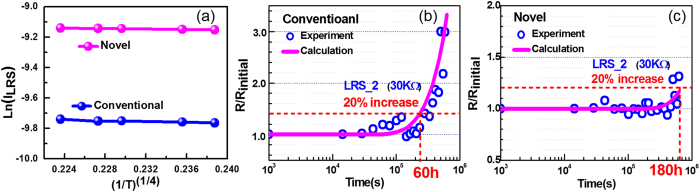
Calculated LRS_2 (fittings of other resistances are not shown here) degradation from the proposed model of the conventional structure and the proposed SEO structure at 150 °C fits well with the experimental data. With the standard of 20% resistance change, the proposed SEO structure shows highly improved retention behavior in comparison with the conventional structure.

Furthermore, since cell resistance is mainly related to the oxygen vacancies distribution, resistance change versus time due to V_O_s diffusion can be derived from V_O_s density evolution versus time. As shown in Fig. 8(a), by characterizing the temperature dependence of LRS for both structures, the conduction mechanism is demonstrated as variable range hopping^21^,^50^, with the relationship between conductivity and V_O_s density as expression (4):


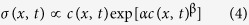


in which, α is calculated as −7 @150 ^o^C corresponding to the measurements, and β is 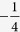 based on the demonstrated variable range hopping conduction mechanism. Accordingly, the change of resistance can be calculated based on the model mentioned as Fig. 7, which fits well with the measured retention data (LRS_2) obtained from both novel and conventional cells (Fig. 8(b,c)), which proves the conclusion that the thermal diffusions of V_O_s is well suppressed in our novel structure even under high temperature and hence dramatically improves the cell retention reliability.

## Conclusion

In this study, remarkable improvements of reliability (endurance: ~10^8^ and retention: ~180 h@150 ^o^C) are achieved by introducing a novel structure of TaO_x_-based 3D vertical RRAM with self-localized switching layer. The newly proposed 3D structure demonstrates excellent self-compliance performance and outstanding cycle-to-cycle and device-to-device uniformity as well. The proposed thermal diffusion model well explains the reliability improvements, and analysis of the experimental data indicate that the spontaneous formation of the self-confined switching region by proposed SEO can effectively inhibit the diffusion of oxygen vacancies as well as suppressing the thermal impacts, indicating that the proposed 3D structure has great potential for ultra-high density non-volatile memory.

## Methods

### Oxidation Optimization

The 2D RRAM devices for optimization experiments are fabricated by the following processes: At first, 100 nm SiO_2_ was deposited on Si wafers by plasma enhanced chemical vapor deposition (PECVD) as isolation layer, and then 170 nm Ta was deposited by PVD and patterned as the bottom electrode (BE). Next, the three samples are annealed in different conditions, i.e. 300 ^o^C/2 h, 400 ^o^C/15 min, 500 ^o^C/30 min. Finally 300 nm PVD Pt was deposited as top electrode (TE).

### Device Fabrication

Detailed fabrication processes of the proposed novel 3D RRAM cell as well as the conventional structure are illustrated as follows: Firstly, 100 nm Si_3_N_4_ was deposited on Si wafers by PECVD as isolation layer, and Ta sidewall electrode (50 nm) was deposited by DC sputtering and patterned by lift-off process respectively as one layer of sidewall electrode. And then, to form a 3D stack, by alternate deposition of Si_3_N_4_/Ta, stacked multilayer thin films were formed, and formation of the via holes was realized by photolithography and dry etching. The above process steps are the same for both structures. Subsequently, in case of the proposed structure, the self-confined switching area was formed by partially oxidizing the Ta sidewall electrode through annealing in oxygen ambient (400 ^o^C/15 min). For a comparison, in this step, a consecutive 15 nm TaO_x_ switching layer was deposited by RF sputtering (Ar: O = 12:8, 500 W) in case of the conventional cell. Finally, 300 nm Pt pillar electrode was formed by DC sputtering and lift-off process for both structure to complete the fabrication.

## Additional Information

**How to cite this article**: Yu, M. *et al.* Novel Vertical 3D Structure of TaO_x_-based RRAM with Self-localized Switching Region by Sidewall Electrode Oxidation. *Sci. Rep.* **6**, 21020; doi: 10.1038/srep21020 (2016).
